# POL7085 or anti‐CCL28 treatment inhibits development of post‐paramyxoviral airway disease

**DOI:** 10.1002/iid3.147

**Published:** 2017-02-02

**Authors:** Becky J. Buelow, Michelle Rohlfing, Françoise Jung, Garry J. Douglas, Mitchell H. Grayson

**Affiliations:** ^1^ Medical College of WisconsinMilwaukeeWisconsinUSA; ^2^ Polyphor LtdAllschwilSwitzerland

**Keywords:** Asthma, chemokines, therapeutics

## Abstract

**Introduction:**

Asthma is major health burden throughout the world, and there are no therapies that have been shown to be able to prevent the development of disease. A severe respiratory paramyxoviral infection early in life has been demonstrated to greatly increase the risk of developing asthma. We have a mouse model of a severe respiratory paramyxoviral infection (Sendai virus, SeV) that mimics human disease, and requires early expression of the cytokine CCL28 to drive the development of post‐viral airway disease. The known receptors for CCL28 are CCR3 and CCR10. However, it is not known if blockade of these receptors will prevent the development of post‐viral airway disease. The objective of this study was to determine if treatment with a protein epitope mimetic antagonist of CCR10, POL7085, will provide sufficient protection against the development of post‐viral airway disease.

**Methods:**

C57BL6 mice were inoculated with SeV or UV inactivated SeV. From day 3–19 post inoculation (PI), mice were subcutaneously administered daily POL7085 or saline, or every other day anti‐CCL28 mAb. On days 8, 10, and 12 PI bronchoalveolar cytokines, serum IgE, and lung cellular constituents were measured. At day 21 PI airway hyper‐reactivity to methacholine and mucous cell metaplasia was measured.

**Results:**

Treatment with either anti‐CCL28 or POL7085 significantly reduced development of airway hyper‐reactivity and mucous cell metaplasia following SeV infection. The prevention of post‐viral airway disease was associated with early reductions in innate immune cells, but did not appear to be due to a reduction in IL‐13 or IgE.

**Conclusions:**

Blockade of CCL28 or CCR10 during an acute severe respiratory paramyxoviral infection is sufficient to prevent the development of post‐viral airway disease. However, the mechanism of action is unclear and requires further exploration.

## Introduction

Asthma is a common, chronic inflammatory disease of the airways that affects all age groups. It is a significant cause of morbidity (missed days of school/work, hospitalizations, decreased quality of life) and mortality. The prevalence of asthma in modern countries continues to be on the rise, but the reason is unclear 1. Effective treatment strategies do exist to provide symptom relief and reduce exacerbations, but there is no known way to prohibit the development of asthma nor is there a known cure for asthma. To develop novel therapeutics to prevent and/or cure asthma, we need to understand the pathogenesis and molecular basis of this complex disease more clearly.

Viral infections have been shown to increase the risk for developing asthma 2. Using the paramyxovirus, murine parainfluenza virus type 1 (Sendai virus; SeV), we have previously demonstrated a mechanistic pathway linking viral respiratory infection with development of post‐viral obstructive lung disease 3. In this murine model, a viral respiratory infection with SeV recruits CD49d^+^ neutrophils to the murine airways, where they induce the expression of the high affinity receptor for IgE (FcϵRI) on lung conventional dendritic cells (cDCs) 4. Crosslinking of antiviral IgE bound to FcϵRI on lung cDCs leads to the production of the chemokine CCL28. CCL28 serves as a chemoattractant to recruit IL‐13 producing Th2 cells to the lung to ultimately drive the development of airway hyper‐responsiveness (AHR) and mucous cell metaplasia (MCM) 3. This SeV‐induced disease is acute as it develops 21 days post‐inoculation (PI). Furthermore, we have shown that CCL28 in the absence of a viral infection is sufficient to drive AHR and MCM 5. Administering an anti‐CCL28 mAb during the viral infection significantly reduced post‐viral MCM and AHR at a late time point (day 49 PI) 5. These data reiterate the importance of CCL28 to the development of post‐viral lung disease in this animal model.

CCL28 or mucosal epithelial chemokine (MEC) has two known receptors, CCR10 and CCR3 6, 7. These chemokine receptors are expressed on eosinophils, Treg cells, Th2 cells, NKT cells, and epithelial cells 7, 8, 9, 10. These are all cell types that have been implicated in the development of allergic rhinitis and allergic airway asthma 8, 11. In fact, the CCL28‐CCR10 axis has been associated with asthma and found to play a significant role in animal models of non‐viral induced asthma 9, 11, 12. In humans with asthma, high levels of CCL28 have been found in their sputum and airways, and we have demonstrated that cross‐linking IgE on human peripheral blood dendritic cells induces increased CCL28 production 6, 13, 14. Therefore, a CCR10 antagonist may serve as a novel therapeutic in the treatment of asthma. Recently we developed a CCR10 antagonist, POL7085, using Protein Epitope Mimetic (PEM) technology, which was shown to significantly decrease allergen‐induced eosinophilic airway inflammation in an OVA‐induced allergic airway inflammation mouse model of asthma 12. Given these results, the primary goal of this study was to assess whether inhibition of CCL28 signaling by the CCR10 antagonist POL7085 would alter development of post‐viral airway disease.

## Methods

### Ethical statement

An ethical statement is not required as there were no human subjects involved in this study.

### Mouse handling

C57BL/6 mice were obtained from The Jackson Laboratory (Bay Harbor, ME). Six week old male mice were used. Mice were housed, handled, and all experiments performed according to protocols approved by the Medical College of Wisconsin Institutional Animal Care and Use Committee. A total of 103 mice were utilized for the experiments detailed in this manuscript.

### Mouse inoculation

Mice were anesthetized using intraperitoneal ketamine and xylazine as previously described 4, 15. Each mouse was inoculated intranasally with 2 × 10^5^ pfu of SeV or ultraviolet light‐inactivated SeV (UV‐ SeV), as we previously published 4, 5, 15.

### Measurement of airway hyper‐responsiveness

To measure AHR a two‐chamber plethysmography system (Buxco Fine Pointe; DSI, New Brighton, MN) was used to measure specific airways resistance (sRaw) and specific airways conductance (sGaw) to increasing doses of aerosolized methacholine in conscious mice, as previously reported 5. Data are presented as percent change from baseline (no methacholine) sRaw or sGaw.

### Histologic evaluation

On day 21 PI with SeV or UV‐SeV, the right lung was removed and fixed in 10% buffered formalin at 25 cm H_2_O pressure, then dehydrated with ethanol and embedded in paraffin. Using Periodic Acid Schiff (PAS), 5 μm thick paraffin sections were stained to determine mucous cells. A blinded observer counted the number of PAS^+^ cells in bronchioles from three random sections per mouse. Data are reported as mean number of PAS^+^ cells/mm BM using ImageJ (NIH) 5, 15.

### Immunoglobulin E (IgE) measurement

Blood was collected on day 8 PI SeV or UV‐SeV. The plasma was removed after mixing with 0.5 mL 10 mM EDTA and centrifuging at 8000 *g* (Eppendorf Centrifuge 5418) for 30 s, and stored at −80°C until analysis. ELISA was used to evaluate total mouse IgE (BioLegend, 432404, sensitivity 0.1 ng/mL) according to the manufacturer's instructions.

### Cytokine analysis

On days 8, 10, and 12 PI SeV or UV‐SeV bronchoalveolar lavage (BAL) was performed with 1 mL PBS × 2. Following centrifugation at 8000 *g* for 30 s, the supernatant was collected and stored at −80°C until evaluated. Using commercial ELISAs the concentration of IL‐9 (eBioscience, 88–8092, sensitivity 32 pg/mL), IL‐10 (BioLegend, 431418, sensitivity 2.7 pg/mL), IL‐13 (eBioscience, BMS6015, sensitivity 2.8 pg/mL), IL‐33 (eBioscience, 88–7333, sensitivity 25 pg/mL), IFNα (eBioscience, BMS6027, sensitivity 7.48 pg/mL), IFNβ (BioLegend, 439407, sensitivity 1.9 pg/mL), and IFN‐γ (eBioscience, BMS606, sensitivity 5.3 pg/mL) in the BAL was determined according to manufacturer's instructions.

### Flow cytometry

#### Cell isolation from lungs

Following BAL, 1 mL of digest media (49.5 mL DMEM‐5 [Dulbecco modified eagle's medium with 5% fetal calf serum] and 0.5 mL HCD mix [hyaluronidase, collagenase, DNase]) was placed into both lungs via cannulated trachea as previously described 3. Each set of lungs was removed, placed in 4 mL of digest media and cut into pieces in a Petri dish on ice. Each set of lungs was then incubated at 37°C in 5% CO_2_ for 45 min. One milliliter of 10 mM EDTA was added and the cells were incubated for an additional 15 min. The pieces of lungs were further dissected and filtered through a 70 μm strainer. The resulting single cell suspension pellet was resuspended in 5 mL of RBC lysing buffer (8.3 g NH_4_Cl + 1 g KHCO_3_ + 0.037 g EDTA in 1 L sterile H_2_0) and incubated for 30 s at room temperature. Five milliliter of 1 × PBS was added and each tube subsequently centrifuged at 2000 rpm for 5 min at 4°C. Supernatant was discarded and the pellet was resuspended in 1 × PBS for flow cytometry.

#### Extracellular staining

Lung immune cell surface markers were stained with mouse antibodies to CD3 (FITC‐labeled clone 17A2, eBioscience), B220 (FITC‐labeled clone RA3‐6B2, eBioscience), CD4 (PE‐labeled clone GK1.5, eBioscience), CD8 (PE‐labeled clone 53–6.7, eBioscience), CD19 (PE‐labeled clone 1D3, eBioscience), CCR10 (APC‐labeled clone 248918, LifeSpan Biosciences), NK1.1 (APC‐labeled clone PK136, eBioscience), CD11c (FITC‐labeled clone N418, eBioscience), Siglec H (FITC‐labeled clone eBio 440c, eBioscience), F4/80 (FITC‐labeled clone BM8, eBioscience), CD11b (PE‐labeled clone M1/70, eBioscience), and FcϵRI (APC‐labeled clone MAR‐1, eBioscience). Appropriate isotype control antibodies were utilized. For each stain, cells were stained in 100 μL of staining buffer (98.3 μL PBS/0.5% BSA + 0.2 μL of Fc block). Each stain was incubated for 20 min on ice in the dark, and washed with 200 μL of PBS/0.5% BSA. The stained cells were then fixed with 200 μL of 1% paraformaldehyde. Cells were evaluated on a Becton–Dickinson LSRII flow cytometer and analyzed with FlowJo software (TreeStar, Ashland, NC).

#### Intracellular staining

Lung immune cell extracellular cytokines were stained with CD3 (PE‐labeled clone 145‐2C11, eBioscience; FITC‐labeled clone 17A2, eBioscience) and CD4 (FITC‐labeled clone RM4‐5, eBioscience; PE‐labeled clone GK1.5, eBioscience).

Lung immune cell intracellular FoxP3 was identified with APC‐labeled clone FJK‐16s, (eBioscience). Appropriate isotype control antibodies were utilized for all stains. Cells were stained extracellularly as above, and then stained intracellular with anti‐Foxp3 or isotype control antibody in 100 μL Triton × buffer + 0.2 μL of Fc block. Cells were resuspended in 200 μL of 1% paraformaldehyde and analyzed as stated above.

### Study design

**Figure 1:**
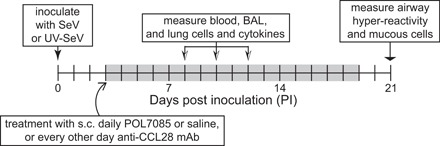
Study design. Mice were inoculated intanasally (i.n.) with either Sendai virus (SeV) or UV inactivated SeV (UV‐SeV) on day 0. From day 3 post inoculation (PI) through day 19 PI (the greyed area in the figure) mice received either daily s.c. POL7085 or saline, or were given s.c. anti‐CCL28 mAb injections every other day. On days 8, 10, and 12 PI blood, BAL, and lung tissue were examined for cell and cytokines (see text for details). On day 21 PI airway hyper‐reactivity was determined, and mice were sacrificed for measurement of mucous cell metaplasia.

The study design is shown in Figure 1. On day 0, mice were inoculated with SeV or UV‐ SeV, and treated with daily subcutaneous (s.c.) 250 μL (15 mg/kg) of Protein Epitope Mimetic (PEM)‐CCR10 antagonist (POL7085; Polyphor Ltd) or 250 μL normal saline or s.c. every other day 100 μg anti‐CCL28 mAb (R&D Systems, #MAB533) starting on day 3 PI SeV/UV‐SeV and continuing until day 19 PI SeV/UV‐SeV. AHR and MCM was determined at day 21 PI SeV/UV‐SeV. For BAL, serum IgE, and cellular data, mice were humanely sacrificed on days 8, 10, and 12 PI SeV/UV‐SeV.

### Statistical analyses

Statistical tests employed included repeated measures and two‐way ANOVA and Student's *t*‐test. Specific tests utilized are mentioned in the figure legends. Data are presented as mean +/− SEM, with significance set at *P *< 0.05.

## Results

### Airway hyper‐reactivity and mucous cell metaplasia

The SeV model has been well characterized, and we have demonstrated that development of post‐viral MCM by day 21 PI requires CCL28 3. In fact, we have also shown that blockade of CCL28 prevents development of both MCM and airway hyper‐reactivity at day 49 PI SEV 5. CCL28 has two known ligands, CCR3 and CCR10, therefore, we undertook this study to determine if CCR10 blockade with a small cyclopeptide antagonist would be capable of preventing the development of post‐viral airway disease.

Mice were inoculated with either SeV or UV inactivated SeV (UV‐SeV) and then treated s.c. with POL7085, a PEM antagonist of CCR10, saline (vehicle), or a mAb against CCL28 as outlined in Figure 1.

**Figure 2:**
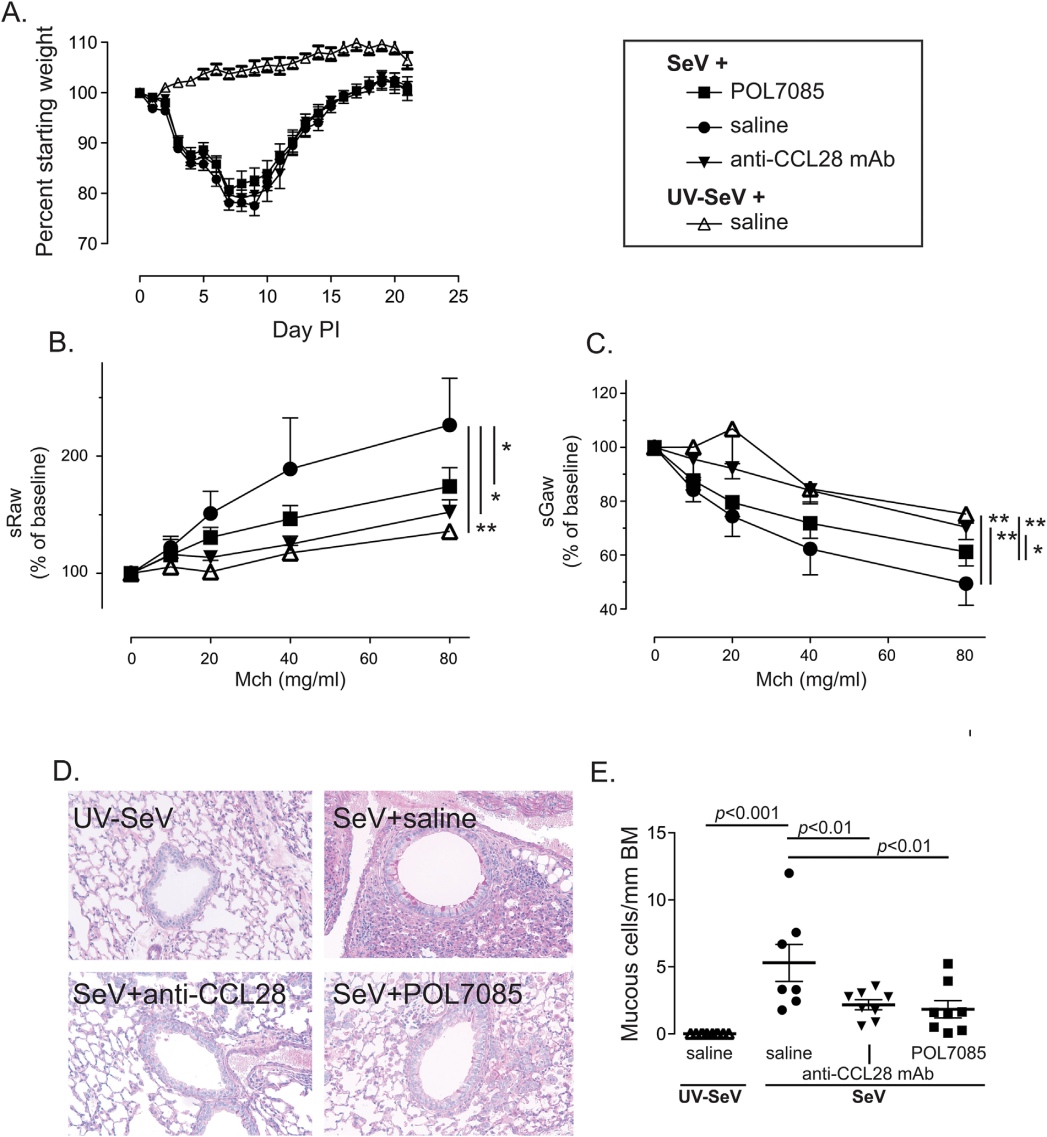
CCL28 or CCR10 blockade inhibit post‐viral airway disease. Male C57BL6 mice were treated as indicated in Figure 1. (A) There was no change in weight loss of SeV infected mice when treated with either POL7085 or anti‐CCL28 mAb. Data from *n* ≥ 9 mice combined from 3 separate experiments. (B) Specific airways resistance (sRaw) and (C) specific airways conductance (sGaw) from mice treated as in Figure 1. Data are from *n* ≥ 6 mice per treatment combined from three separate experiments. (D) Representative periodic acid Schiff (PAS) stained sections (200×) from mice treated as in Figure 1. (E) Quantification of PAS^+^ cells per millimeter of basement membrane (BM). Data are from *n* ≥ 7 mice per treatment combined from three separate experiments. For B, C, and E, repeated measures ANOVA with Newman–Keuls multiple comparison post test was used with *P < 0.05, **P < 0.01.

Treatment with anti‐CCL28 mAb or POL7085 had no obvious physical effects on the mice. In fact, as shown in Figure 2A, these treatments had no effect on the severe weight loss that is a component of the SeV infection model 3. However, by day 21 PI treatment with either therapy the development of post‐viral airway disease was markedly reduced, as evidenced by a significantly reduced increase in sRaw and decrease in sGaw to methacholine challenge when compared to the SeV infected saline control group (Figs. 2B and C).

The other major hallmark of the post‐viral airway disease that develops in the SeV model is MCM. As shown in Figures 2D and E, treatment with either anti‐CCL28 mAb or POL7085 both significantly reduced the development of mucous cells by day 21 PI SeV. Therefore, antagonizing either CCL28 or CCR10 is sufficient to prevent the development of post‐viral airway disease by day 21 PI.

### Cytokines and IgE

**Figure 3:**
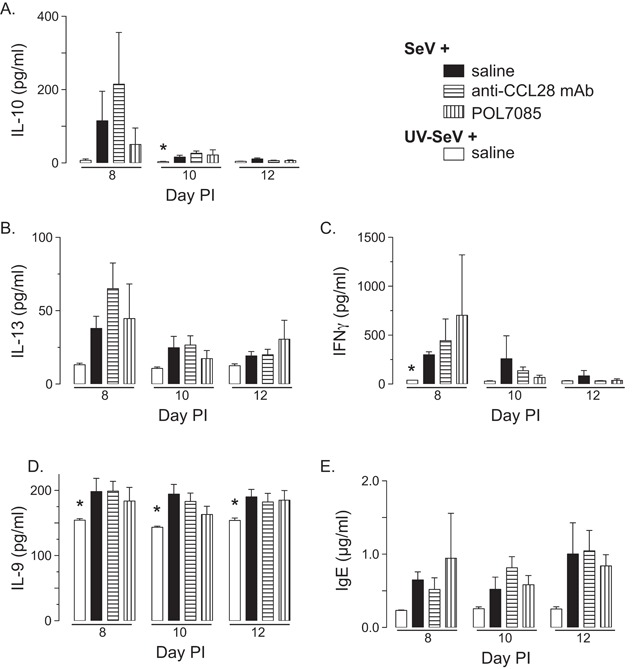
Effect of CCL28 or CCR10 antagonism on airway cytokines and serum IgE. (A) IL‐10, (B) IL‐13, (C) IFNγ, and (D) IL‐9 measured from the bronchoalveolar lavage (BAL) of mice treated as in Figure 1 on days 8, 10, and 12 PI. (E) Total serum IgE on days 8, 10, and 12 PI from mice treated as in Figure 1. Data from *n* ≥ 2 mice per treatment per day, and from samples run in duplicate. Statistical comparisons were done with unpaired Student's *t*‐test (*P < 0.05 vs. SeV + saline).

SeV is cleared from the airways between day 10–12 PI 3. Therefore, we hypothesized that treatment with anti‐CCL28 or POL7085 would alter the cytokines being produced during the viral infection. To test this, we obtained BAL from mice on days 8, 10, and 12 PI and measured the concentrations of IL‐10, IL‐13, and IFNγ. We chose IL‐10 as it is a regulatory cytokine that could help dampen the immune response and subsequent post‐viral airway disease. IL‐13 was chosen, as it has been shown to drive post‐viral airway hyper‐reactivity and MCM in this model 16, 17. As shown in Figure 3A–C, treatment with anti‐CCL28 or POL7085 led to no change in the concentrations of BAL IL‐10, IL‐13, or IFNγ. Therefore, blockade of CCL28/CCR10 does not prevent development of post‐viral airway disease through a global reduction of IL‐13–at least not at days 8–12 PI.

Other cytokines that have been implicated in asthma include IL‐9 and IL‐33 18, 19. Therefore, we examined whether blockade of CCL28/CCR10 had any effect on these cytokines in the BAL. As shown in Figure 3D, while IL‐9 levels were elevated with viral infection, they were not affected by any of the treatments. Further, IL‐33 was undetectable in any of the samples (data not shown). Therefore, the effects of blockade of CCL28/CCR10 do not seem to be mediated through either IL‐9 or IL‐33.

One hallmark of SeV induced post‐viral airway disease is that it depends upon increased production of IgE and expression of the high‐affinity receptor for IgE (FcϵRI) on dendritic cells 3. Therefore, we examined whether treatment with POL7085 or a mAb against CCL28 had an effect on total serum IgE. As shown in Figure 3D, there was no effect on total IgE levels with any of the treatments.

### Cellular composition

**Figure 4:**
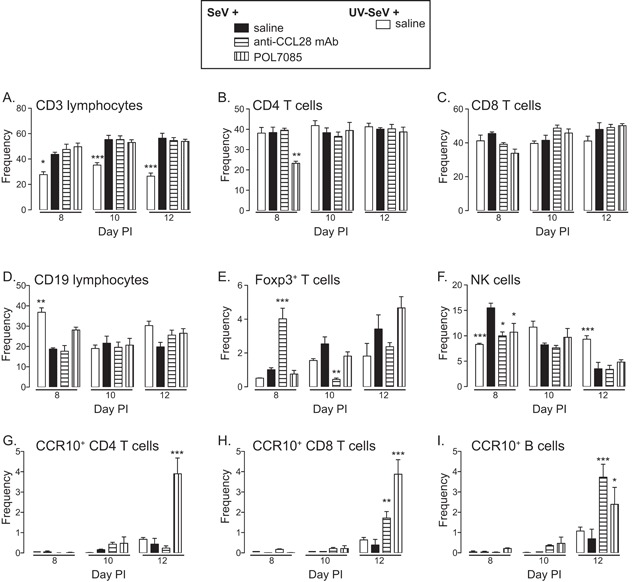
Lymphocytic cellular inflammation during paramyxoviral infection. Mice were treated as indicated in Figure 1, and then at day 8, 10, and 12 PI the cellular composition of the lung parenchyma was determined by flow cytometry. (A) Frequency of CD3 expressing lymphocytes (T cells). (B) Frequency of CD3^+^ lymphocytes expressing CD4 (CD4 T cells). (C) Frequency of CD3^+^ lymphocytes expressing CD8 (CD8 T cells). (D) Frequency of CD19 expressing lymphocytes (B cells). (E) Frequency of CD3^+^CD4^+^ lymphocytes expressing intracellular Foxp3 (percent of Tregs in CD4^+^ T cells). (F) NK cells as identified by the frequency of lymphocytes expressing NK1.1. The frequency of (G) CD4 T cells, (H) CD8 T cells, or (I) B cells expressing CCR10. Data are from *n* ≥ 4 mice per time point and condition. Two‐way ANOVA with Bonferonni multiple comparisons post‐test comparing groups to SeV + saline. *P < 0.05, **P < 0.01, ***P < 0.001.

Since, our data did not demonstrate an obvious effect on lung cytokines, we next assessed whether the treatments had an effect on the lung cellular infiltrate during the viral infection. At various days PI SeV or UV‐SeV we used flow cytometry to characterize the lymphocytic inflammatory cells in the lung parenchyma. As shown in Figure 4A, infection with SeV led to increased frequency of CD3^+^ T cells in the lung, however, this increase was unaffected by any of the treatments. Interestingly, treatment with POL7085 led to an early, but not sustained, reduction in the frequency of CD4^+^ T cells (Fig. 4B) compared to the other treatments. There was no effect on CD8^+^ T cells with any treatment (Fig. 4C). Similarly, B cell frequency was unaffected by any treatment (Fig. 4D).

While, there was some variability in the frequency of regulatory T cells (Foxp3^+^ T cells), the overall percentage of these cells was quite small and did not seem to correlate with the treatments (Fig. 4E). One cell type that was reduced with both CCL28 and CCR10 antagonism was NK cells (Fig. 4F); however, this effect was modest and limited to only day 8 PI.

Since, none of the changes in lung lymphocyte subsets seemed to correlate with antagonism of CCL28 or CCR10, we next hypothesized that these treatments would impair recruitment of CCR10 expressing T and/or B cells to the lung. After verifying that treatment with POL7085 did not interfere with our ability to detect CCR10 expression by flow cytometry (supplemental figure), we determined the frequency of CD4^+^ T cells, CD8^+^ T cells, and B220^+^/CD19^+^ B cells that expressed CCR10 at various days PI (Fig. 4 G–I respectively). Interestingly, treatment with POL7085 significantly increased the frequency of all three cell types by day 12 PI, while blockade of CCL28 led to a small increase in CD8 T cells and a more robust increase in B cells at day 12 PI. Thus, treatment with anti‐CCL28 or CCR10 antagonist led to a small but significant increase in CCR10 expressing lymphocytes in the lung. It is tempting to hypothesize that this increase was due to inhibition of a CCL28/CCR10 mediated migration of lymphocytes out of the lung parenchyma and into the BAL. However, we did not analyze the cellular component of the BAL, so this remains only speculative.

### Dendritic subsets and macrophages

**Figure 5:**
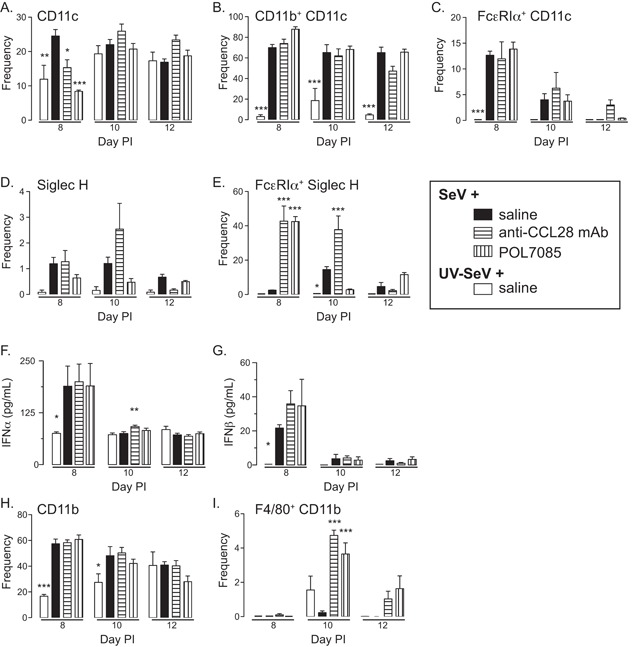
Effect of CCL28 or CCR10 antagonism on dendritic cell and macrophages subsets during a paramyxoviral infection. Mice were treated as in Figure 1 and on days 8, 10, and 12 PI lung cellular inflammation characterized by flow cytometry. (A) Frequency of conventional dendritic cells (cDC) determined as the percent of CD11c expressing cells in the granulocytic gate. (B) Frequency of CD11b expression on CD11c cDC. (C) Frequency of expression of the high‐affinity receptor for IgE, FcϵRIα, on cDC. (D) Frequency of plasmacytoid dendritic cells (pDC) as identified by Siglec H expressing cells. (E) Frequency of pDC expressing FcϵRIα. (F) IFNα and (G) IFNβ levels in BAL of mice as treated in Figure 3. (H) CD11b expressing cells, and (I) the frequency of these cells that co‐expressed F4/80. Data are from *n* ≥ 3 mice per time point and condition. (A–E and H, I) Two‐way ANOVA with Bonferonni multiple comparisons post‐test or (F and G) unpaired Student's *t*‐test comparing groups to SeV + saline. *P < 0.05, **P < 0.01, ***P < 0.001.

We have shown previously that SeV drives expression of FcϵRI on lung cDC and plasmacytoid dendritic cells (pDC) 3. And, as mentioned, expression of FcϵRI on cDC is critical for development of post‐viral airway disease. Therefore, we examined the dendritic cell subsets in the airways of mice at days 8, 10, and 12 PI, to see if treatment with anti‐CCL28 or POL7085 altered their presence in the airways. As shown in Figure 5A, both anti‐CCL28 and POL7085 reduced the frequency of cDC in the airways at day 8 PI SeV, with POL7085 having a greater effect. However, similar to the effect on CD4^+^ T cells, this reduction was only transient, with levels being similar amongst all treatments at days 10 and 12 PI. In terms of cDC subsets, we found no difference in lung cDC expression of CD11b or FcϵRI, although both were clearly induced by SeV infection (Fig. 5B and C).

In contrast to the cDC, pDC levels were unaffected by any treatment (Fig. 5D), but blockade of CCL28 or CCR10 led to a significant and marked increase in the frequency of these cells that expressed FcϵRI at day 8 PI SeV (Fig. 5E). This increase was sustained to day 10 in anti‐CCL28 treated mice, but not in the POL7085 treated mice. Increased FcϵRI expression on pDC has been associated with a reduced type‐I IFN response in humans, but whether a similar situation occurs in mice is not known, nor does it likely provide an explanation for the efficacy of the treatments in blocking post‐viral airway disease. Nonetheless, we measured IFNα and IFNβ levels in the BAL at days 8, 10, and 12 PI. As shown in Figure 5F and G, infection with SeV led to increased IFNα and β at day 8 PI, but the various treatments had no appreciable effect on these levels. At day 10 PI SeV there was a significant but very small increase in IFNα levels in mice treated with anti‐CCL28 mAb; however, this difference is unlikely to explain the prevention of post‐viral airway disease in this group.

Finally, we wanted to determine if either of the treatments had any effect on any other myeloid cell or parenchymal macrophages. Therefore, we examined the frequency of CD11b expressing cells in the lung at days 8, 10, and 12 PI. While there was an increase in CD11b expressing cells with SeV infection at day 8 PI, blockade of CCL28 or CCR10 failed to have any effect (Fig. 5F). While all myeloid cells express CD11b, macrophages can be identified as expressing F4/80 in addition to CD11b 20. As shown in Figure 5G, treatment with anti‐CCL28 or POL7085 led to a marked increase in macrophages in the lung at day 10 PI SeV, which was waning by day 12. Again, as with the other cellular data, whether this simply reflects variability in the model or all relates to the reduction in development of post‐viral airway disease is unclear.

## Discussion

Asthma represents a major health burden, and one etiology for the development of asthma is a severe paramyxoviral infection early in life 2. We have a well‐characterized murine model that utilizes the paramyxovirus, Sendai virus. With this model we have demonstrated the importance of IgE and CCL28 3, 5. Given that CCL28 has two known ligands, we hypothesized in this study that a PEM antagonist of CCR10 would have equal efficacy as CCL28 at preventing the development post‐viral airway disease.

Our hypothesis is based upon the assumption that human airway disease develops in a similar manner as that seen in our mouse model. It is important to stress that the studies that have found CCL28 elevated in the airways or sputum of asthma patients have not demonstrated a causal link—only an association 6, 13. Further, our studies of cross‐linking IgE on human dendritic cells demonstrated an increase in CCL28 production, but unlike mouse dendritic cells, human dendritic cells were already producing CCL28 at baseline 14. An additional difference between mouse and man is the fact that mouse dendritic cells lack expression of FcϵRI at baseline, while human cells have been shown to express this receptor from birth 21. Therefore, the general transferability of the findings in mice to the human has not been well established.

In this report, we demonstrate that treatment with the CCR10 antagonist POL7085, early in an acute respiratory viral infection is sufficient to prevent development of post‐viral airway hyper‐reactivity and MCM. This effect was equivalent to that seen with anti‐CCL28 mAb treatment. This would suggest that the effect of CCL28 in this model of post‐viral airway disease is through CCR10 and not CCR3, the other receptor for CCL28. We previously showed that infection with SeV led to an increase in CCR10 expressing lymphocytes in the respiratory mucosa, while another group demonstrated that CCR3 expressing cells are not increased with SeV infection 3, 22. Therefore, in this model, we believe that the effects of CCL28 are mediated through CCR10. However, because we were unable to demonstrate any specific mechanism to explain the successful prevention of post‐viral airway disease, we cannot discount that the CCR10 antagonist acted through other pathways potentially unrelated to CCL28.

In our model, CCL28 is produced at or shortly after the peak of weight loss 3. Therefore, it is not surprising that treatment with either anti‐CCL28 or a CCR10 antagonist failed to have any effect on the weight loss due to SeV infection. This suggests that the effect of these treatments are unrelated to the mechanisms that drive weight loss during a severe paramyxoviral infection.

Our model is dependent upon the production of IgE against virus, followed by expression of FcϵRI on lung cDC. Crosslinking FcϵRI drives production of CCL28, which is required for development of post‐viral disease. However, treatment with either anti‐CCL28 or POL7085 had no appreciable effect on either total IgE or expression of FcϵRI on cDC. Interestingly, pDC expressed FcϵRI did increase significantly with treatment, but the relevance of this effect is unclear. In humans, crosslinking FcϵRI on pDC leads to impaired production of type I IFN (IFNα/β) 23. Our data, however, demonstrate no evidence that treatment with anti‐CCL28 or POL7085 reduced the amount of type I IFN being produced during the anti‐viral immune response, at least in the BAL. As with cytokines, it is possible that there was an effect on lung parenchymal type I IFN production, or effects at earlier time points than we studied. These possibilities await further investigation.

Development of post‐viral airway disease has been shown to depend upon expression of IL‐13 in the airways 16, 17. Again, we thought that the efficacy of the anti‐CCL28 and/or CCR10 antagonist treatment would be due to reduction in IL‐13 in the airways. However, we found no difference in IL‐13 levels, nor was there an increase in the concentration of the regulatory cytokine IL‐10. It is possible that BAL cytokine levels might not correlate with lung parenchymal levels, as we did not measure the level of IL‐13 in the parenchyma.

We noted several changes in cell frequencies with anti‐CCL28 and CCR10 antagonism. However, most of these changes were transient and at very early time‐points in the infection. In particular, NK cells and cDC were all significantly reduced at day 8 PI SeV with treatment, but by day 10 PI all were no different from the SeV + saline control. It may well be that the effect of these therapies are primarily at time points even earlier than day 8 PI. We have focused on days 8–12 PI because the adaptive immune response in the lung develops around day 6 PI, and we thought the effect of these therapies would be on the adaptive response 24. Given that we also saw an effect on pDC (FcϵRI expression) and macrophages (increased at day 10 PI SeV), it seems more likely that the effects of anti‐CCL28 and CCR10 antagonism is via the innate and not adaptive response. Further, studies will need to be performed to better understand this effect.

Finally, it is important to note that in these studies we did not utilize an IgG control for the anti‐CCL28 treatment. This was done to avoid the use of extra mice, when we had already published that blockade of CCL28 (with appropriate IgG control groups) inhibited the development of post‐viral MCM at day 21 PI SeV and both MCM and airway hyper‐reactivity at day 49 PI SeV (treated from day 14–49) 3, 5. Therefore, we felt that the addition of the extra control group was unwarranted for this study, as the anti‐CCL28 mAb group really represented a “positive” control for efficacy of the treatment.

In this report, we demonstrate that treatment with either an anti‐CCL28 mAb or the CCR10 PEM antagonist POL7085 prevented the acute development of post‐paramyxoviral airway disease in a mouse model. The mechanism underlying this prevention appears not to be due to a simple reduction in IL‐13, but may involve multiple innate cells early in the viral infection. Whether treatment with a CCR10 antagonist after the development of disease would also ameliorate the airway hyper‐reactivity and MCM was not studied, but this is something that could have significant clinical implications if it does work. Future, studies will be focused on understanding the mechanism of these therapies and their utilities as potential therapeutics in the treatment of post‐viral obstructive lung disease.

## Conflicts of Interest

None declared.
